# Diffuse White Matter Signal Abnormalities on Magnetic Resonance Imaging Are Associated With Human Immunodeficiency Virus Type 1 Viral Escape in the Central Nervous System Among Patients With Neurological Symptoms

**DOI:** 10.1093/cid/cix035

**Published:** 2017-03-13

**Authors:** Ruthiran Kugathasan, Dami A. Collier, Lewis J. Haddow, Kate El Bouzidi, Simon G. Edwards, Jonathan D. Cartledge, Robert F. Miller, Ravindra K. Gupta

**Affiliations:** 1 University College London Hospital NHS Foundation Trust, United Kingdom; 2 Division of Infection and Immunity, University College London, United Kingdom; 3 Central and North West London NHS Foundation Trust, United Kingdom; 4 Research Department of Infection and Population Health, University College London, United Kingdom; 5 Department of Clinical Research, Faculty of Infectious and Tropical Diseases, London School of Hygiene and Tropical Medicine, United Kingdom

**Keywords:** HIV, viral escape, neurocognitive impairment, CSF, reservoir.

## Abstract

**Background.:**

Human immunodeficiency virus type 1 (HIV-1) can replicate independently in extravascular compartments such as the central nervous system, resulting in either cerebrospinal fluid (CSF) discordance (viral load [VL] in CSF 0.5 log_10_ copies HIV-1 RNA greater than plasma VL) or escape (detection of HIV VL >50 copies/mL in CSF in patients with suppressed plasma VL <50 copies/mL). Both discordance and escape may be associated with neurological symptoms. We explored risk factors for CSF discordance and escape in patients presenting with diverse neurological problems.

**Methods.:**

HIV-infected adult patients undergoing diagnostic lumbar puncture (LP) at a single center between 2011 and 2015 were included in the analysis. Clinical and neuroimaging variables associated with CSF discordance/escape were identified using multivariate logistic regression.

**Results.:**

One hundred forty-six patients with a median age of 45.3 (interquartile range [IQR], 39.6–51.5) years underwent 163 LPs. Median CD4 count was 430 (IQR, 190–620) cells/µL. Twenty-four (14.7%) LPs in 22 patients showed CSF discordance, of which 10 (6.1%) LPs in 9 patients represented CSF escape. In multivariate analysis, both CSF discordance and escape were associated with diffuse white matter signal abnormalities (DWMSAs) on cranial magnetic resonance imaging (adjusted odds ratio, 10.3 [95% confidence interval {CI}, 2.3–45.0], *P* = .007 and 56.9 [95% CI, 4.0–882.8], *P* = .01, respectively). All 7 patients with CSF escape (10 LPs) had been diagnosed with HIV >7 years prior to LP, and 6 of 6 patients with resistance data had documented evidence of drug-resistant virus in plasma.

**Conclusions.:**

Among patients presenting with diverse neurological problems, CSF discordance or escape was observed in 15%, with treatment-experienced patients dominating the escape group. DWMSAs in HIV-infected individuals presenting with neurological problems should raise suspicion of possible CSF discordance/escape.

## Summary

Among patients presenting with diverse neurological problems at a single center, cerebrospinal fluid discordance or escape was observed in 15% and was associated with diffuse white matter signal abnormalities on cranial magnetic resonance imaging.

There is a growing body of evidence that human immunodeficiency virus (HIV) can continue to replicate in disparate compartments during suppressive antiretroviral therapy (ART). Up to 10% of individuals on ART who are peripherally suppressed have detectable HIV RNA in cerebrospinal fluid (CSF) [1–5]. Reasons for this include intermittent adherence and treatment interruption where viral rebound in the plasma may be quicker to suppress than in the CSF [3]. There are also data on inadequate control in CSF due to regimens of suboptimal potency, for example, protease inhibitor (PI) monotherapy, or triple therapy with drug resistance [4, 6–8]. In a significant proportion, however, the cause of discordance is unclear and possibilities include lower penetration of ART into the central nervous system (CNS) with subsequent subtherapeutic CSF drug levels [9].

A clinical penetration effectiveness (CPE) score attempted to classify ART penetration into the CNS based on pharmacological properties, measurable CSF drug concentrations, and evidence of reduction in CSF viral load (VL) or improvement in cognition in response to treatment [10]. Rawson et al [1] and Cusini et al [11] found HIV CSF discordance to be associated with lower CPE scores. Canestri et al [4] conducted a retrospective cohort study of 11 patients with CSF discordance and found that in the majority, HIV in CSF had developed resistance mutations. Furthermore, neurological symptoms and CSF VL improved upon changing to a regimen with a higher CPE score. A more recent prospective study by Nightingale et al [2] found that CSF discordance was significantly associated with low-level peripheral HIV type 1 (HIV-1) viremia and nadir CD4 count; no association was observed between CSF ART concentration or CPE scores.

Detectable HIV in the CSF is hypothesized to increase the risk of neurological syndromes and neurocognitive impairment (NCI) [4, 12, 13]. The Multicenter AIDS Cohort Study [14] found the incidence of HIV dementia among men who have sex with men to be 21% between 1990 and 1992, which decreased to 10% between 1996 and 1998 following the introduction of 3-drug combination ART (cART) [14]. An elevated HIV-1 CSF VL compared to plasma has been associated with NCI [15] and dementia in untreated patients [16]. More recently, the presence of HIV in CSF was associated with an inflammatory response [17] and low-level peripheral viremia in cART-treated patients [17]. Two case series [4, 12] have identified cases of CSF discordance with neurological symptoms.

Despite an increasing understanding of CSF discordance, there are gaps in knowledge, for example, identification of risk factors associated with development of CSF discordance and an understanding of which patients would benefit from further investigation in a clinical setting. In particular, data that assess the association between a patient’s neurological presenting complaint and magnetic resonance imaging (MRI) results with CSF discordance are lacking, and it is these 2 factors that most strongly lead the clinician to perform a lumbar puncture (LP). We retrospectively studied a population of HIV-infected individuals who underwent an LP for a clinical indication, to identify risk factors associated with HIV CSF/plasma discordance and CSF escape.

## METHODS

Participants were selected using records from a single laboratory serving a population of 3800 HIV-infected individuals in 2013, the median year of study. By 2015, the number had risen to 4500, and 95% of these patients were on ART, with 90% having plasma VL <50 copies/mL. The inclusion criteria for the study were as follows: (1) adults living with HIV over the age of 18 having a clinically indicated LP at Mortimer Marker and University College London Hospital; and (2) LP performed during the period 27 October 2011 to 9 April 2015. The following LP events were excluded: (1) a repeat LP within 90 days of the first; (2) LPs where the plasma HIV VL was measured >30 days from the time of the LP, or no plasma VL was measured; (3) LPs where the MRI was done >90 days from the date of the LP.

Using hospital and clinic electronic databases, data were collected on the following characteristics: age, sex, HIV VL in plasma and CSF at the time of LP (within 30 days), CD4 count within 30 days of LP, MRI brain findings within 180 days of LP, ART regimen at the time of the LP, CPE scores for the ART regimen, and clinical indication for the LP (LP within 30 days of clinical assessment) (Supplementary Tables 1 and 2).

Indications for LP were grouped into acute neurology, chronic symptoms of NCI, and subacute neurological complaint. Acute neurology was defined as new neurological presentation including headaches, confusion, sensory and motor signs, and symptoms within 1 month of onset. Chronic symptoms of NCI included global cognitive impairment, memory impairment, attention difficulties, and cognitive impairment with neuropathy, and also included follow-up consultations for a previous neurocognitive problem. The subacute neurological complaint group included any other neurological presentation (Supplementary Table 2).

ART regimens were divided into those containing a nucleoside reverse transcriptase inhibitor (NRTI), a nonnucleoside reverse transcriptase inhibitor (NNRTI), a ritonavir-boosted PI, an integrase inhibitor, and/or a CCR5 inhibitor (Supplementary Figure 1). CPE scores were calculated using the scoring system devised by Letendre and colleagues [10]. Discordance was defined as a HIV-1 VL in the CSF compartment that was 0.5 log_10_ copies HIV-1 RNA greater than the VL in peripheral blood, based on criteria used in similar studies [1, 2]. CSF escape was defined by the presence of a detectable CSF HIV VL (>50 copies per mL) in patients with an undetectable peripheral blood VL (<50 copies per mL).

MRI findings were derived from clinical reports created by a specialist neuroradiologist, at the time of the patient’s presentation. The neuroradiologist was blinded to whether patients met criteria for discordance or escape. MRIs were categorized as showing volume loss, diffuse white matter signal abnormality (DWMSA), focal white matter lesion (FWML), or other abnormalities. DWMSA was further graded as subtle or definite on the basis of qualitative information provided in the report. Definite DWMSA was defined as the presence of white matter changes involving multiple regions of both supra- and infratentorial white matter [18]. The presence of definite DWMSA was supported by increased water diffusivity on the apparent diffusion coefficient map, or was reported as consistent with viral encephalitis. DWMSA was categorized as subtle if it was described as subtle or ill-defined. Small, focal white matter lesions of presumed vascular origin were categorized as FWML and were not included in the definition of DWMSA. T2-hyperintense white matter lesions consistent with progressive multifocal leukoencephalopathy or other opportunistic infections were categorized as other MRI abnormalities, as were space-occupying lesions, hemorrhage, and infarct.

Statistical analyses were done using Stata software version 13 (StataCorp, College Station, Texas). Univariate analyses were performed to identify factors associated with discordance and CNS escape independently. Factors with a *P* value of <.1 were included in a multivariable logistic regression. A random- effects model was used to control for the effect of clustering as some participants underwent multiple LPs during the observation period. We did not perform statistical analysis for an association between ART regimen and CNS discordance/escape due to concerns regarding confounding.

## RESULTS

Of 194 LPs performed, 163 LPs from 146 individuals were included in the present analysis (Supplementary Figure 2). Baseline characteristics of the study population are shown in Table 1. Median age was 45.3 (interquartile range [IQR], 39.6–51.5; range, 25.1–79.2) years. The majority (70.5%) of LPs were done in men. Median CD4 cell count at the time of LP was 430 (IQR, 190–620) cells/µL, with a median nadir CD4 count of 130 (IQR, 50–260) cells/µL. Of LPs, 80.8% (126/156) were done in patients on ART (7 missing) (Table 1). Of these, 39 (30.9%) did not have VL < 50 copies/mL in plasma. CSF discordance was observed in 24 (15%) of LPs in 22 patients and viral escape in CSF observed in 10 LPs (6%) in 9 patients.

**Table 1.** Baseline Characteristics for Cerebrospinal Fluid Samples and Individuals

Characteristic	Overall Denominator, No.	No. (%)
Male sex	163	115 (70.5)
Median age at time of LP, y (IQR)	163	45.3 (39.6–51.5)
Total No. of LPs	163	
No. of LPs per individual		
1		132 (90.4)
2		12 (8.2)
3		1 (0.7)
4		1 (0.7)
CNS viral escape	163	10 (6.1)
CNS CSF/plasma discordance	163	24 (14.7)
Presenting symptoms	163	
Acute neurology		48 (29.4)
Chronic symptoms of neurocognitive impairment		61 (37.4)
Subacute neurological complaint		44 (27.0)
Missing information		5 (3.1)
Follow-up of an earlier problem		5 (3.1)
Focal neurological signs	151/163	18 (11.9)
Status	163	
Unsuppressed in plasma but no discordance		60 (36.8)
Discordant and plasma VL detectable		14 (8.6)
Escape, plasma VL undetectable		11 (6.8)
Suppressed in both CSF and plasma		78 (47.8)
Median CSF VL, log_10_ copies/mL (IQR)	163	1.7 (1.7–3.1)
Median plasma VL, log_10_ copies/mL (IQR)	163	1.7 (1.7–3.8)
Median nadir CD4, cells/µL (IQR)	122/163	130 (50–260)
Median current CD4, cells/µL (IQR)	159/163	430 (190–620)
Undetectable CSF VL	163	98 (60.1)
Undetectable plasma VL	163	90 (55.2)
On ART (n = 126)	156/163	126 (80.8)
On standard ART (3 drugs, 2 classes)		75 (59.5)
ART regimen containing NRTI		104 (82.5)
ART regimen containing NNRTI		31 (24.6)
Combination PI therapy		75 (59.5)
ART regimen containing integrase inhibitor		10 (7.9)
ART regimen containing CCR5 inhibitor		7 (5.6)
ART regimen solely PI monotherapy		16 (12.7)
CPE score (n = 126)	156/163	
Low (<7)		48 (38.1)
Medium (7)		51 (40.5)
High (>7)		27 (21.4)
Focal MRI white matter lesions	136/163	42 (30.9)
Diffuse MRI white matter lesions	136/163	
Nil		84 (61.8)
Subtle		38 (28.0)
Definite		14 (10.2)
Parenchymal volume loss	136/163	41 (30.2)
Other pathological MRI findings	136/163	35 (25.7)

Data are presented as No. (%) unless otherwise indicated; no./No. are shown where there are missing data.

Abbreviations: ART, antiretroviral therapy; CNS, central nervous system; CPE, clinical penetration effectiveness; CSF, cerebrospinal fluid; IQR, interquartile range; LP, lumbar puncture; MRI, magnetic resonance imaging; NK, not known; NNRTI, nonnucleoside reverse transcriptase inhibitor; NRTI, nucleoside reverse transcriptase inhibitor; PI, protease inhibitor; VL, viral load.

More than one-third of patients presented with chronic symptoms indicative of NCI. The remaining patients were split fairly evenly between acute neurological presentations and subacute presentations (Supplementary Table 1). MRI reports from 141 records were available and 5 were excluded as they were performed >6 months from the date of the LP. The median time between MRI and LP was 7 (IQR, 2–42) days. Among LPs, 11.9% (18/151) were done in the context of focal neurological signs.

Neither CSF discordance nor CSF escape was associated with age, sex, presenting symptoms, or CPE score. In multivariate analysis, CSF discordance was associated with DWMSA on MRI (adjusted odds ratio [aOR], 10.3 [95% confidence interval {CI}, 2.3–45.0]; *P* = .007) (Table 2). There was no evidence of clustering of the data in the logistic regression with random-effects modeling (data not shown), suggesting that there was no impact of multiple LPs from the same patient on the statistical analyses. Results were not significantly altered when only patients on ART at the time of LP were considered (data not shown).

**Table 2.** Factors Associated With Human Immunodeficiency Virus Cerebrospinal Fluid Discordance (n = 163 Lumbar Punctures)

Characteristic			Univariable Analysis		Multivariable Analysis	
	Discordant CSF/Plasma		Unadjusted Odds Ratio (95% CI)	*P* Value^a^	Adjusted Odds Ratio (95% CI)	*P* Value^b^
	No (n = 139)	Yes (n = 24)				
Male sex: No. of LPs	98 (71)	17 (71)	1.0 (.4–2.6)	.97		
Male sex	90 (70)	11 (61)	0.7 (.2–1.9)	.43		
No. of LPs by age, y						
23–38	35 (25)	3 (13)	1	.16		
39–44	33 (24)	7 (29)	2.5 (.6–10.6)			
45–51	41 (30)	6 (25)	1.7 (.4–7.4)			
52–80	30 (22)	8 (33)	3.1 (.7–13.2)			
No. of LPs by presenting symptoms						
Acute neurology	42 (32)	6 (27)	1	.97		
Chronic symptoms of NCI	36 (28)	8 (36)	1.6 (.5–5.0)			
Subacute neurological complaint (n/N = 153/163)	53 (41)	8 (36)	1.1 (.3–3.3)			
Total No. of LPs						
1	116 (84)	16 (67)	1	.006	1	.04
2	20 (14)	4 (17)	1.5 (.4–4.8)		1.9 (.5–7.2)	
≥3	3 (2)	4 (17)	9.7 (1.8–50.7)		30.4 (2.1–446.0)	
Median plasma VL, log_10_ copies/mL (IQR)	1.7 (1.7–3.8)	2.1 (1.7–3.8)		.55^c^		
Median nadir CD4, cells/µL (IQR) (n/N = 122/163)	130 (40–250)	152 (50–260)		.76^c^		
No. of LPs by nadir CD4 category, cells/µL (n/N =						
122/163)						
0–99	37 (36)	7 (37)				
100–199	30 (29)	5 (26)				
>200	36 (35)	7 (37)				
Median current CD4 count, cells/µL (IQR) (n/N = 159/163)	430 (180–620)	445 (330–770)				
On ART, No. of LPs (n/N = 156/163)	105 (79)	21 (91)				
No. of LPs by CPE category						
No ART	28 (21)	2 (09)	1	.66		
Low	37 (28)	11 (48)	4.2 (.8–21.3)			
Medium	46 (35)	5 (22)	1.5 (.3–8.5)			
High	22 (17)	5 (22)	3.2 (.5–18.8)			
Using medium CPE as baseline						
Off ART			0.8 (.2–4.0)			
Low			3.1 (.8–11.9)			
Medium			1 (base)			
High			1.9 (.4–8.9)			
Focal MRI white matter lesions, No. of LPs	36 (31)	6 (29)	0.9 (.3–2.5)	.80		
Diffuse white matter, No. of LPs						
Nil	79 (66)	10 (48)	1	.02	1	.007
Subtle	33 (28)	5 (24)	1.1 (.4–3.6)		1.4 (.4–5.4)	
Definite	8 (7)	6 (29)	5.6 (1.5–20.6)		10.3 (2.3–45.0)	

Data are presented as No. (%) unless otherwise indicated; no./No. are shown where there are missing data.

Abbreviations: ART, antiretroviral therapy; CI, confidence interval; CPE, clinical penetration effectiveness score; CSF, cerebrospinal fluid; IQR, interquartile range; LP, lumbar puncture; MRI, magnetic resonance imaging; NCI, neurocognitive impairment; VL, viral load.

a χ^2^ test.

b Multiple logistic regression.

c Wilcoxon rank-sum test.

DWMSA on MRI (aOR, 56.9 [95% CI, 3.9–882.8], *P* = .003) was also associated with CSF escape in multivariate analyses (Table 3). The association between DWMSA and escape was still present when the acute MRIs were excluded from analysis (data not shown). Patients with CSF escape had been diagnosed at least 7 years prior to LP, and had received previous ART regimens, with evidence of drug resistance in plasma associated virus in 6 of 6 individuals who had resistance tests sent (Table 4).

**Table 3.** Factors Associated With Human Immunodeficiency Virus Cerebrospinal Fluid Escape (n = 88 Lumbar Puncture Episodes)

Characteristic	CSF/Plasma Viral Escape		Univariable Analysis		Multivariable Analysis	
	No (n = 78)	Yes (n = 10)	Unadjusted Odds Ratio (95% CI)	*P* Value^a^	Adjusted Odds Ratio (95% CI)	*P* Value^b^
Male sex, No. of LPs	59 (75.6)	7 (70.0)	0.8 (.2–3.2)	.70		
No. of LPs by age, y						
23–38	16 (20.5)	1 (10)	1	.46		
39–44	21 (26.9)	3 (30)	2.3 (.2–25.2)			
45–51	22 (28.2)	3 (30)	2.2 (.2–23.9)			
52–80	19 (24.4)	3 (30)	2.5 (.2–28.1)			
No. of LPs by presenting symptoms						
Acute neurology	21 (27.6)	2 (20)	1	.45		
Chronic symptoms of NCI	20 (26.3)	2 (20)	1.1 (.1–8.4)			
Subacute neurological complaint (n/N = 86/88)	35 (46.1)	6 (60)	1.8 (.3–9.9)			
Median nadir CD4, cells/µL (IQR) (n/N = 64/88)	152 (90–250)	230 (25–260)		.64^c^		
No. of LPs by nadir CD4 category, cells/µL						
0–99	15 (26.3)	2 (28)		.51		
100–199	20 (35.1)	1 (14)				
>200 (n/N = 64/88)	22 (38.6)	4 (57)				
Median current CD4, cells/µL (IQR) n/N = (87/88)	520 (370–710)	470 (420–600)		.87^c^		
On ART	75 (97.4)	10 (100)		…		
CPE category, No. of LPs						
Low	28 (36)	3 (30)	1	.38		
Medium	32 (41)	3 (30)	0.9 (.2–4.8)			
High	18 (23)	4 (40)	2.1 (.4–10.7)			
Focal MRI white matter lesions (n/N = 28/76)	24 (36)	4 (40)	1.2 (.3–4.6)	.83		
Diffuse white matter, No. of LPs						
Nil	49 (74)	4 (40)	1	.003	1	.01
Subtle	15 (23)	3 (30)	2.5 (.5–12.5)		2.7 (.4–17.9)	
Definite (n/N = 76/88)	3 (3)	3 (30)	18.4 (1.8–188.6)		56.9 (4.0–822.8)	

Data are presented as No. (%) unless otherwise indicated; no./No. are shown where there are missing data.

Abbreviations: ART, antiretroviral therapy; CI, confidence interval; CPE, clinical penetration effectiveness score; CSF, cerebrospinal fluid; IQR, interquartile range; LP, lumbar puncture; MRI, magnetic resonance imaging; NCI, neurocognitive impairment.

a χ^2^ test.

b Multiple logistic regression.

c Wilcoxon rank-sum test.

**Table 4.** Information on Individuals with Cerebrospinal Fluid Escape (n = 7)

Age at LP, y	Year of HIV Diagnosis	CD4 at LP, Cells/ µL	Nadir CD4, Cells/µL	Year of LP	Sex	ART at Time of LP	Resistance Mutations in Plasma Virus (Year Detected)	Interpretation of Mutation
44	2000	980	260	2014	M	RAL, MVC, DRV/r	Protease: L10I/V (2014)	Reduced PI susceptibility
33	2004	600	152	2011	F	ATV/r, TDF	RT: M184V (2004)	High level resistance to 3TC and FTC
							RT: K103N (2004)	Reduced susceptibility to EFV and NVP
51	2004	440		2012	M	ATV/r, ABC/3TC/ZDV	RT: K103N (2004)	Reduced susceptibility to EFV and NVP
55	1996	420	20	2013	M	DRV/r, RAL, MVC	RT: L210W (2011)	ZDV resistance
							RT: T215Y (2011)	Intermediate/high-level resistance to ZDV and d4T, low-level resistance to ddI, and potentially low-level resistance to ABC and TDF.
							RT: V108IV (2011)	Associated with NRTI resistance
							RT: Y181C (2011)	Reduced susceptibility to NNRTI
41	1995	490	25	2013	F	DRV/r, RAL, TDF	RT: V75I (2001)	
							RT: Q151M (2001)	High-level resistance to ZDV, d4T, ddI, and ABC
							RT: D67N (2001)	
							RT: K70N (2001)	
42	2000	1360		2012	F	LPV/r, TDF	RT: 67N 70R 215Y (2007)	ZDV/ddI resistance
							RT: M184V (2007)	
							RT: K219EK (2007)	
50	1997	450		2014	F	Off ART 08/2014	Resistance testing not performed	
						ABC/ZDV/3TC, DRV/r		

Abbreviations: 3TC, lamivudine; ABC, abacavir; ART, antiretroviral therapy; ATV/r, ritonavir-boosted atazanavir; d4T, stavudine; ddI, didanosine; DRV/r, ritonavir-boosted darunavir; EFV, efavirenz; F, female; FTC, emtricitabine; HIV, human immunodeficiency virus; LP, lumbar puncture; LPV/r, ritonavir-boosted lopinavir; M, male; MVC, maraviroc; NNRTI, nonnucleoside reverse transcriptase inhibitor; NRTI, nucleoside reverse transcriptase inhibitor; NVP, nevirapine; PI, protease inhibitor; RAL, raltegravir; RT, reverse transcriptase; TDF, tenofovir disoproxil fumarate; ZDV, zidovudine.

## DISCUSSION

In this single-center retrospective study, 15% of individuals undergoing LP had CSF/plasma VL discordance and 6% had CNS escape. The 15% prevalence of CSF discordance is similar to the 12% reported by Nightingale et al [2] and the 21% found by Rawson et al in London [1]. The 6% prevalence of CSF escape agrees with the 0–15% quoted in a systematic review by Pérez Valero et al [5].

In patients on ART, CSF discordance was observed in 8%. We found DWMSA on MRI to be significantly associated with CSF discordance and escape, and this is a novel finding. Diffuse white matter signal abnormalities were found in all 11 of a small, uncontrolled, cohort of patients with CSF discordance [4], and in the majority of another retrospective cohort study [12]. Diffuse white matter signal abnormalities occur more commonly in the HIV-infected population compared with the HIV-uninfected population and become more extensive with duration of infection [18]. The presence of HIV in the CSF is proinflammatory as evidenced by raised levels of CSF neopterin [3] and cytokine profiles [17]. Nightingale et al [17], using CSF samples from the penetration of antiretroviral therapy into the nervous system study, showed the presence of higher levels of inflammatory cytokines in the CSF of individuals with discordance when compared with matched controls. The presence of diffuse white matter signal abnormalities on MRI is associated with neurocognitive deficit. Neuropathological correlation suggests that these changes can be caused by HIV replication [19].

In the subgroup analysis, individuals with CSF escape had normal (>350 cells/µL) CD4 counts at the time of the LP. This observation is consistent with findings from the case series by Canestri et al [4] and Peluso et al [12]. This highlights that the presence of a normal CD4 count does not exclude the possibility of CSF escape.

We found no association with CPE scores agreeing with the recent study by Nightingale et al [2], but in contrast to the findings of both Rawson et al [1] and Canestri et al [4]. Given that ART regimen was not random and likely a confounding factor, this result for CPE score should be viewed with caution.

The limitations associated with this study mostly relate to its cross-sectional nature. The lack of association with CPE score could be confounded by potential prescriber bias. In addition, we did not measure patient compliance with ART or record the start dates of ART therapy. There is a very short decay period between when ART is initiated or changed and the CSF VL starts to fall [20]. The indications for MRI were not blinded, and could have resulted in reporter bias with more acute presentations less likely to elicit comments on subtle findings such as white matter changes. The impact of this is likely small, since the association is still present when the acute MRIs are excluded from analysis. A further limitation is that we did not have data on HIV drug resistance in CSF, patients’ use of recreational drugs, or their ethnicity and sexuality.

Of note, our study was not able to explore the effect of PI monotherapy on CNS discordance/escape. In the context of adequate viral load monitoring, PI monotherapy can be used in some individuals with reduced toxicity and no increase in cross-resistance [21–23], despite randomized controlled trials showing inferior suppression of HIV-1 in CSF with boosted PI monotherapy compared with triple therapy [6, 24]. There are retrospective reports of patients developing NCI on boosted PI monotherapy with evidence of CSF discordance [7, 25], although the protease inhibitor therapy versus ongoing triple therapy trial showed no evidence for risk of neurological symptoms in suppressed patients who were randomized to boosted PI monotherapy, compared to those continuing standard triple therapy [26].

In summary, this study adds to the body of knowledge by demonstrating an association between MRI diffuse white matter signal abnormalities and both HIV discordance and escape. Previous studies of discordance and MRI findings have involved smaller numbers and have not directly measured the strength of the association. Physicians should be aware that the presence of DWMSAs on MRI in a peripherally suppressed individual with neurological symptoms may suggest HIV replication in CSF and that a normal CD4 count does not exclude this phenomenon. Longitudinal studies with serial MRI findings correlated with peripheral and CSF VL will be needed to further evaluate this association. Finally, we advocate the formation of collaborative cohorts in order to increase power to detect risk factors for virus escape in CSF under specific clinical presentations, as has been recently proposed [27].

## Supplementary Material

Supplementary Data

## Notes

***Acknowledgments.*** We thank the Department of Virology, University College London Hospitals NHS Foundation Trust, London and Mortimer Market Centre, Central and North West London NHS Foundation Trust, London, for assistance with searching electronic patient records.

***Author contributions*.** Study concept: R. K. G. and R. K.; study design: R. K. G., R. K., R. F. M., L. J. H., J. D. C., S. G. E.; data collection: R. K., L. J. H., and K. E. B.; data analysis: L. J. H. and D. A. C.; data interpretation: R. K. G., R. K., R. F. M., L. J. H., K. E. B., D. A. C., S. G. E., J. D. C.; writing of the manuscript: R. K. G., R. K., R. F. M., L. J. H., K. E. B., D. A. C., S. G. E., J. D. C.

***Financial support*.** This work was supported by the Wellcome Trust (fellowships WT093722MA to R. K. G. and 170461 to K. E. B.), and the British Infection Society (fellowship E19115 to D. A. C.). We also acknowledge support from the National Institute for Health Research, University College London Hospitals Biomedical Research Centre.

***Potential conflicts of interest*.** R. F. M. has received honoraria from Gilead, Janssen, Merck, and ViiV for giving nonpromotional lectures on clinical aspects of HIV; is a member of the British HIV Association Tuberculosis/HIV Guidelines Committee; and is a panel member for the Guidelines for the Prevention and Treatment of Opportunistic Infections in HIV-Infected Adults and Adolescents (National Institutes of Health, Centers for Disease Control and Prevention, HIV Medicine Association of the Infectious Diseases Society of America). J. D. C. has received honoraria as an advisory board consultant, education consultant, and lecturer by Gilead, and as a lecturer by Janssen and an advisory board consultant and lecturer for BMS. R. K. G. has received honoraria from Janssen for giving nonpromotional lectures on HIV drug resistance. All other authors report no potential conflicts. All authors have submitted the ICMJE Form for Disclosure of Potential Conflicts of Interest. Conflicts that the editors consider relevant to the content of the manuscript have been disclosed.
