# Supplementary material for: Diffuse White Matter Signal Abnormalities on Magnetic Resonance Imaging Are Associated With Human Immunodeficiency Virus Type 1 Viral Escape in the Central Nervous System Among Patients With Neurological Symptoms
Source: Clin Infect Dis. 2017 Mar 13;64(8):1059–65. doi: 10.1093/cid/cix035 (PMC5439343; doi:10.1093/cid/cix035)
Supplement: Supplementary Data [file cix035_Supplementary_Data.zip › 84579_Table_S1.pdf]

Table S1. Summary of presenting complaints: Focal neurology was defined as neurological deficit affecting a particular location or function such as arm weakness, leg numbness or speech deficit. Complex neurology was defined as any neurological syndrome not classified into the other categories.

**Acute neurology**	**Headache +/- other neurological symptoms**	**17**
	**Paralysis, focal neurology or similar**	**12**
	**Confusional state**	**12**
	**Severe neuropsychiatric or behavioural symptoms**	**5**
	**Complex neurology including seizures**	**2**
	**Follow-up of earlier acute illness**	**2**
	**Coma**	**1**
**Chronic symptoms of neurocognitive impairment (NCI)**	Global cognitive impairment, not otherwise specified	50
	Memory symptoms	11
	Attention / concentration difficulties	2
	Cognitive impairment with neuropathy	2
	Follow-up of previously diagnosed NCI	1
**Sub acute neurological complaint**	Ataxia	5
	Non-severe neuropsychiatric or behavioural change	4
	Bradykinesia	2
	Chronic or recurrent headaches	18
	Falls +/- cognitive impairment or other neurology	1
	Investigation of previous collapse / LOC	4
	Follow-up of other illness (neurosyphilis, cryptococcosis, lymphoma)	4
	Dizziness	1
	Dysphasia, dysarthria	2
	Hydrocephalus	2
	Peripheral neuropathy	3
	Multiple neurological complaints	1
	Seizures	9
	Tremor	2
