# Supplementary material for: Diffuse White Matter Signal Abnormalities on Magnetic Resonance Imaging Are Associated With Human Immunodeficiency Virus Type 1 Viral Escape in the Central Nervous System Among Patients With Neurological Symptoms
Source: Clin Infect Dis. 2017 Mar 13;64(8):1059–65. doi: 10.1093/cid/cix035 (PMC5439343; doi:10.1093/cid/cix035)
Supplement: Supplementary Data [file cix035_Supplementary_Data.zip › 84579_Figure_S1.pdf]

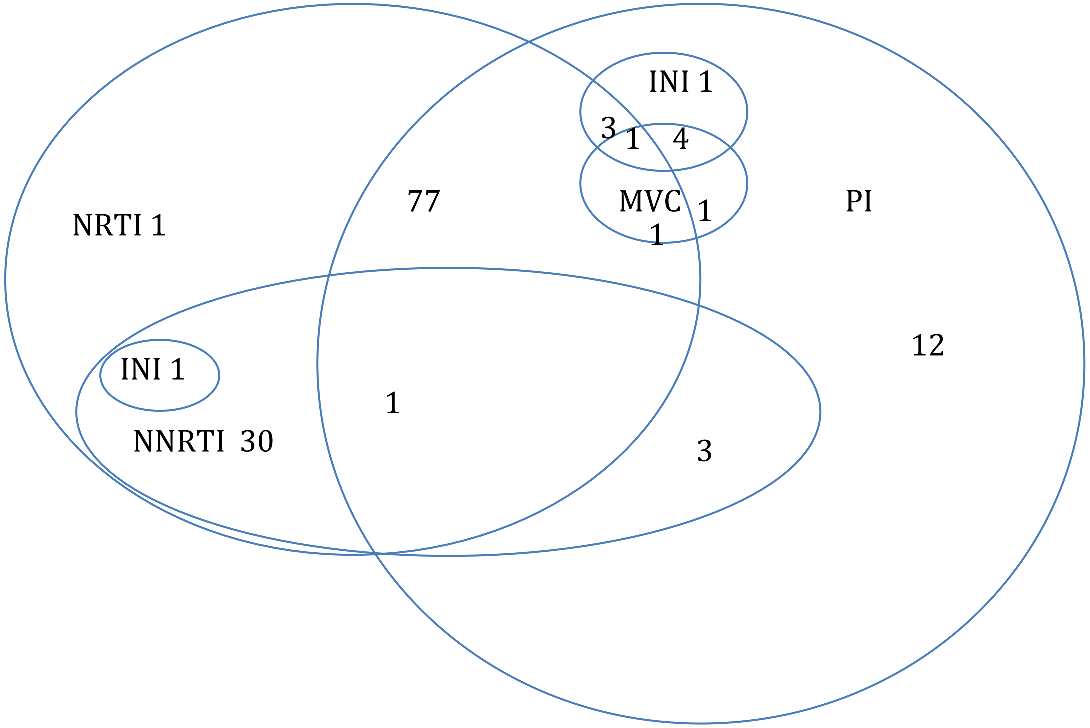

Figure S1: Venn diagram showing antiretroviral drug treatments at the time of LP. In 42 patients ARV regimen was not specified, or they were not taking ARVs.
Key: PI = protease inhibitor; NRTI = nucleoside reverse transcriptase inhibitor; NNRTI = non-nucleoside reverse transcriptase Inhibitor; INI = integrase inhibitor; MVC = maraviroc.
