# Supplementary figures and images for: Diffuse White Matter Signal Abnormalities on Magnetic Resonance Imaging Are Associated With Human Immunodeficiency Virus Type 1 Viral Escape in the Central Nervous System Among Patients With Neurological Symptoms
Source: Clin Infect Dis. 2017 Mar 13;64(8):1059–65. doi: 10.1093/cid/cix035 (PMC5439343; doi:10.1093/cid/cix035)

**Figure S2: Flow diagram for participant and LP selection**

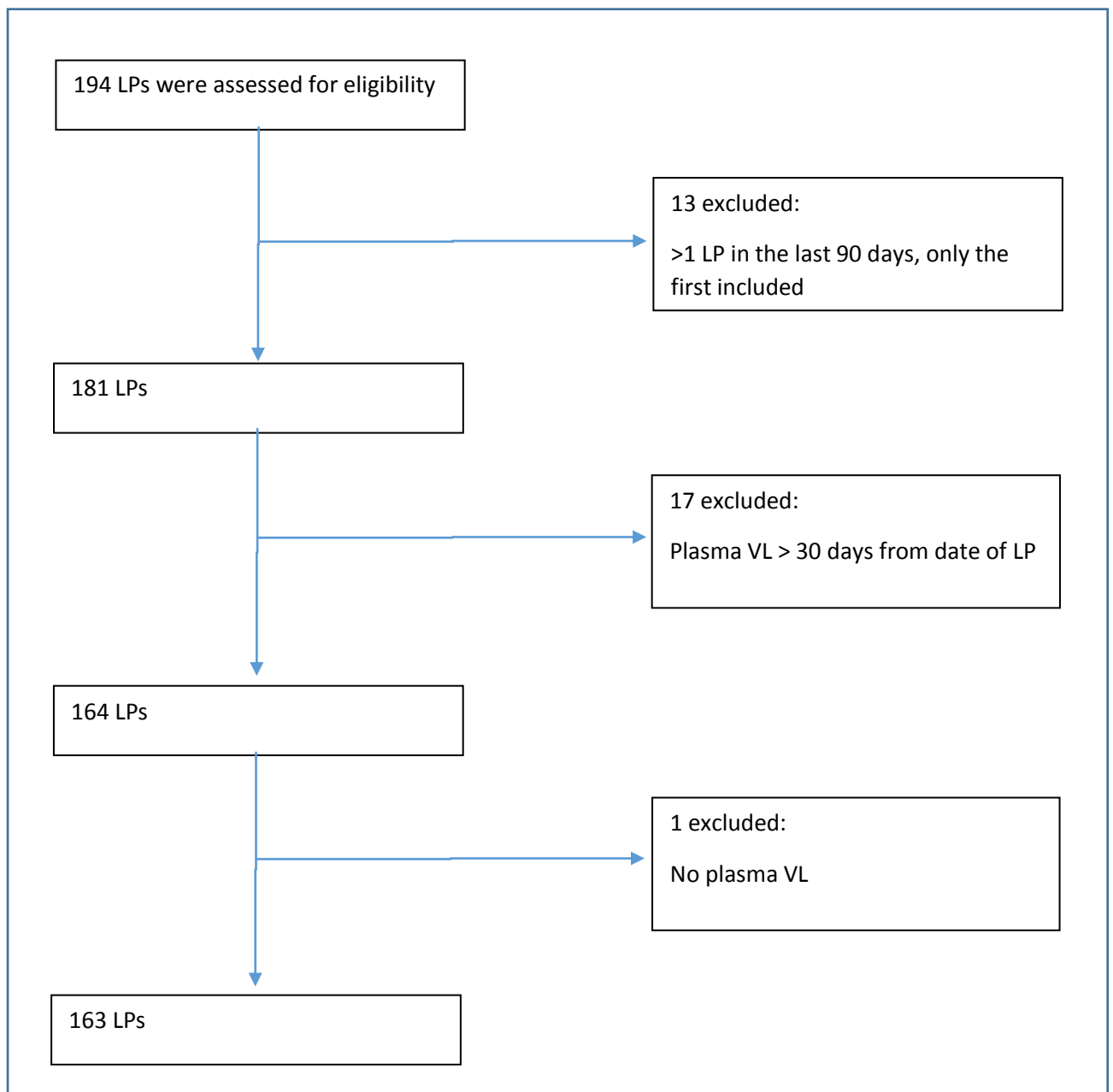

Supplement: Supplementary Data [file cix035_Supplementary_Data.zip › 84579_Figure_S2.pdf]
